# *N*-(1,3-Thia­zol-2-yl)-*N*′-[(thio­phen-2-yl)carbon­yl]thio­urea hemihydrate

**DOI:** 10.1107/S160053681204500X

**Published:** 2012-11-07

**Authors:** Durga Prasad Singh, Seema Pratap, Sema Öztürk Yildirim, Ray J. Butcher

**Affiliations:** a Department of Chemistry, M.M.V., Banaras Hindu University, Varanasi 221 005, India; b Erciyes University, Faculty of Sciences, Department of Physics, 38039 Kayseri, Turkey; c Department of Chemistry, Howard University, 525 College Street NW, Washington, DC 20059, USA

## Abstract

The title compound, C_9_H_7_N_3_OS_3_·0.5H_2_O, crystallizes with two independent but similar mol­ecules in the asymmetric unit, both of which are linked by a water mol­ecule through O—H⋯N hydrogen bonds. In addition the water O atom is further linked by N—H⋯O hydrogen bonds to two additional main mol­ecules, forming a tetra­meric unit. These tetra­meric units then form infinite ribbons parallel to the *ac* plane.The dihedral angle between the thio­phenoyl and thia­zolyl rings is 12.15 (10) and 21.69 (11)° in mol­ecules *A* and *B*, respectively. The central thio­urea core makes dihedral angles of 5.77 (11) and 8.61 (9)°, respectively, with the thio­­phen­oyl and thia­zolyl rings in mol­ecule *A* and 8.41 (10) and 13.43 (12)° in mol­ecule *B*. Each mol­ecule adopts a *trans–cis* geometry with respect to the position of thio­phenoyl and thia­zole groups relative to the S atom across the thio­urea C—N bonds. This geometry is stabilized by intra­molecular N—H⋯O hydrogen bonds.

## Related literature

For general background to aroyl­thio­urea and its derivatives, see: Aly *et al.* (2007 ▶). For related structures, see: Koch (2001 ▶); Pérez *et al.* (2008 ▶). For their biological activity, see: Saeed *et al.* (2008 ▶); Gu *et al.* (2007 ▶); Xu *et al.* (2004 ▶); Yan & Xue (2008 ▶). 
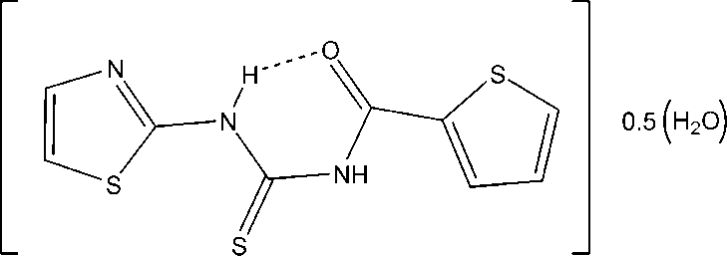


## Experimental

### 

#### Crystal data

C_9_H_7_N_3_OS_3_·0.5H_2_O
*M* *_r_* = 278.37
Triclinic, 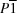

*a* = 7.4489 (4) Å
*b* = 11.1060 (6) Å
*c* = 14.7935 (7) Å
α = 93.559 (4)°
β = 99.813 (4)°
γ = 107.789 (5)°
*V* = 1139.74 (11) Å^3^
*Z* = 4
Cu *K*α radiation
μ = 5.86 mm^−1^
*T* = 123 K
0.35 × 0.25 × 0.18 mm

#### Data collection

Oxford Diffraction Xcalibur (Ruby, Gemini CCD) diffractometer
Absorption correction: multi-scan (*CrysAlis PRO*; Agilent, 2011 ▶) *T*_min_ = 0.441, *T*_max_ = 1.000
7828 measured reflections
4566 independent reflections
3906 reflections with *I* > 2σ(*I*)
*R*_int_ = 0.034

#### Refinement

*R*[*F* ^2^ > 2σ(*F* ^2^)] = 0.039
*wR*(*F* ^2^) = 0.110
*S* = 1.08
4566 reflections
304 parameters
3 restraints
H atoms treated by a mixture of independent and constrained refinement
Δρ_max_ = 0.43 e Å^−3^
Δρ_min_ = −0.30 e Å^−3^

### 

Data collection: *CrysAlis PRO* (Agilent, 2011 ▶); cell refinement: *CrysAlis PRO*; data reduction: *CrysAlis PRO*; program(s) used to solve structure: *SHELXS97* (Sheldrick, 2008 ▶); program(s) used to refine structure: *SHELXL97* (Sheldrick, 2008 ▶); molecular graphics: *SHELXTL* (Sheldrick, 2008 ▶); software used to prepare material for publication: *SHELXTL*.

## Supplementary Material

Crystal structure: contains datablock(s) I, global. DOI: 10.1107/S160053681204500X/zs2240sup1.cif

Structure factors: contains datablock(s) I. DOI: 10.1107/S160053681204500X/zs2240Isup2.hkl

Supplementary material file. DOI: 10.1107/S160053681204500X/zs2240Isup3.cml

Additional supplementary materials:  crystallographic information; 3D view; checkCIF report

## Figures and Tables

**Table 1:** Hydrogen-bond geometry (Å, °)

*D*—H⋯*A*	*D*—H	H⋯*A*	*D*⋯*A*	*D*—H⋯*A*
N1*A*—H1*AA*⋯O1*W* ^i^	0.86	2.22	3.003 (3)	152
N1*B*—H1*BA*⋯O1*W* ^ii^	0.86	2.14	2.973 (3)	163
N2*A*—H2*AA*⋯O1*A*	0.86	1.89	2.599 (3)	138
N2*B*—H2*BA*⋯O1*B*	0.86	1.90	2.588 (3)	136
O1*W*—H1*W*⋯N3*B*	0.82 (1)	2.06 (1)	2.852 (3)	163 (4)
O1*W*—H2*W*⋯N3*A*	0.82 (1)	2.09 (1)	2.892 (3)	167 (3)

Symmetry codes: (i) 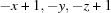; (ii) 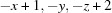.

**Fig. 1.**
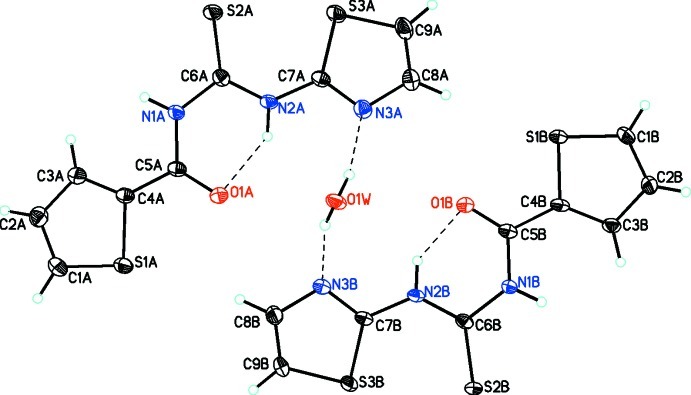
Molecular structure of the title compound showing the atom labeling scheme and 30% probability displacement ellipsoids. Dashed lines indicate the intramolecular N—H···O and inter-species O—H···N hydrogen bonds.

**Fig. 2.**
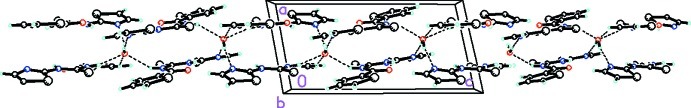
Crystal packing for the title compound viewed along the c axis. Dashed lines indicate an intermolecular N—H···O and O—H···N hydrogen bonds.

## supplementary crystallographic information

### Comment

Aroylthiourea and its derivatives are an important class of organic compounds in which the sulphur atom is a major ligand atom and plays an important role in coordination chemistry with transition metals. These compounds are found to be useful in heterocyclic synthesis and many of these substrates have interesting biological activities (Aly *et al.*, 2007). Aroylthioureas and and its derivatives are also known to exhibit a wide range of biological activities, such as anticancer (Saeed *et al.*, 2010), anti-fungal (Saeed *et al.*, 2008), antibacterial, antiviral, anti-tubercular, insecticidal, organocatalyst (Gu *et al.*, 2007) and as agrochemicals (Xu *et al*., 2004).

The title compound (Fig. 1), C_9_H_7_N_3_OS_3_.0.5H_2_O, crystallizes with two independent but similar molecules in the asymmetric unit both of which are linked by a water molecule through O—H···N hydrogen bonds. In addition the water O is further linked by N-H···O hydrogen bonds to two additional C_9_H_7_N_3_OS_3_ molecules, forming a tetrameric moiety. These tetrameric moieties then form infinite ribbons parallel to the ac plane (Fig.2).

The main bond lengths and angles are within the range obtained for similar compounds (Koch *et al.*, 2001; Perez *et al.*, 2008). The C6A-S2A [1.657 (2)Å], C6B-S2B [1.659 (2)Å] and C5A-O1A [1.233 (3)Å], C5B-O1B [1.232 (3)Å] bonds show typical double-bond character. However, the C-N bond lengths, C5A-N1A [1.388 (3)Å], C6A-N1A [1.395 (3)Å], C6A-N2A [1.345 (3)Å], C7A-N2A [1.383 (3)Å] and C5B-N1B [1.385 (3)Å], C6B-N1B [1.390 (3)Å], C6B-N2B [1.350 (3)Å], C7B-N2B [1.383 (3)Å] are shorter than the normal C-N single-bond length of about 1.48 Å (Allen, 2002). These results can be explained by the existence of resonance in this part of the molecule. In first molecule(A) the central thiourea fragment (N1A-C6A-S2A-N2A) makes the dihedral angle of 5.77(0.11)° and 8.61(0.09 )° with thiophenoyl (S1A/C4A-C1A) and thiazolyl ring (C7A-S3A-C9A-C8A-N3A). Where as in second molecule(B) the central thiourea fragment (N1B/C6B/S2B/N2B) makes the dihedral angle of 8.41(0.10)° with (S1B/C4B-C1B) group, and the thiazole ring (C7B-S3B-C9B-C8B-N3B) is 13.43(0.12)°, respectively. The dihedral angle between the thiophenoyl and thiazolyl rings is 12.15(0.10)° in molecule A and 21.69(0.11)° in molecule B. The trans-cis geometry in the thiourea moiety of both molecule is stabilized by the N-H···O and C-H···O hydrogen bonds (Fig.2 and Table 1).

### Experimental

A solution of 2-thiophenecarbonyl chloride (0.01 mol) in anhydrous acetone (80 ml) was added dropwise to a suspension of ammonium thiocyanate (0.01 mol) in anhydrous acetone (50 ml) and the reaction mixture was refluxed for 50 minutes. After cooling to room temperature, a solution of 4-chloroaniline (0.01 mol) in dry acetone (25 ml) was added and the resulting mixture refluxed for 2 h. The reaction mixture was poured into five times its volume of cold water, upon which the thiourea precipitated. The product was recrystallized from ethanol as colorless block crystals.

### Refinement

Hydrogen atoms on the water molecule were located in a difference-Fourier map and both positional and isotropic displacement parameters were refined. Other H atoms were placed in calculated positions with N—H = 0.88 Å and C—H = 0.95 Å and refined using a riding model, with *U*_iso_(H) = 1.2*U*_eq_(C, N).

### Crystal data

**Table:**

C_9_H_7_N_3_OS_3_·0.5H_2_O	*Z* = 4
*M**_r_* = 278.37	*F*(000) = 572
Triclinic, *P*1	*D*_x_ = 1.622 Mg m^−^^3^
Hall symbol: -P 1	Cu *K*α radiation, λ = 1.54184 Å
*a* = 7.4489 (4) Å	Cell parameters from 3735 reflections
*b* = 11.1060 (6) Å	θ = 3.1–75.6°
*c* = 14.7935 (7) Å	µ = 5.86 mm^−^^1^
α = 93.559 (4)°	*T* = 123 K
β = 99.813 (4)°	Block, colorless
γ = 107.789 (5)°	0.35 × 0.25 × 0.18 mm
*V* = 1139.74 (11) Å^3^	

### Data collection

**Table:**

Oxford Diffraction Xcalibur (Ruby, Gemini CCD) diffractometer	4566 independent reflections
Radiation source: Enhance (Cu) X-ray Source	3906 reflections with *I* > 2σ(*I*)
Graphite monochromator	*R*_int_ = 0.034
Detector resolution: 10.5081 pixels mm^-1^	θ_max_ = 75.8°, θ_min_ = 3.1°
ω scans	*h* = −9→6
Absorption correction: multi-scan (*CrysAlis PRO*; Agilent, 2011)	*k* = −13→13
*T*_min_ = 0.441, *T*_max_ = 1.000	*l* = −12→18
7828 measured reflections	

### Refinement

**Table:**

Refinement on *F*^2^	Primary atom site location: structure-invariant direct methods
Least-squares matrix: full	Secondary atom site location: difference Fourier map
*R*[*F*^2^ > 2σ(*F*^2^)] = 0.039	Hydrogen site location: inferred from neighbouring sites
*wR*(*F*^2^) = 0.110	H atoms treated by a mixture of independent and constrained refinement
*S* = 1.08	*w* = 1/[σ^2^(*F*_o_^2^) + (0.0566*P*)^2^ + 0.1264*P*] where *P* = (*F*_o_^2^ + 2*F*_c_^2^)/3
4566 reflections	(Δ/σ)_max_ = 0.001
304 parameters	Δρ_max_ = 0.43 e Å^−^^3^
3 restraints	Δρ_min_ = −0.30 e Å^−^^3^

### Special details

**Table:**

Geometry. All e.s.d.'s (except the e.s.d. in the dihedral angle between two l.s. planes) are estimated using the full covariance matrix. The cell e.s.d.'s are taken into account individually in the estimation of e.s.d.'s in distances, angles and torsion angles; correlations between e.s.d.'s in cell parameters are only used when they are defined by crystal symmetry. An approximate (isotropic) treatment of cell e.s.d.'s is used for estimating e.s.d.'s involving l.s. planes.
Refinement. Refinement of *F*^2^ against ALL reflections. The weighted *R*-factor *wR* and goodness of fit *S* are based on *F*^2^, conventional *R*-factors *R* are based on *F*, with *F* set to zero for negative *F*^2^. The threshold expression of *F*^2^ > σ(*F*^2^) is used only for calculating *R*-factors(gt) *etc*. and is not relevant to the choice of reflections for refinement. *R*-factors based on *F*^2^ are statistically about twice as large as those based on *F*, and *R*- factors based on ALL data will be even larger.

### Fractional atomic coordinates and isotropic or equivalent isotropic displacement parameters (Å^2^)

**Table:**

	*x*	*y*	*z*	*U*_iso_*/*U*_eq_	
S1A	0.17373 (9)	0.25490 (5)	0.47025 (4)	0.02167 (15)	
S1B	0.26874 (10)	−0.44204 (6)	0.98233 (4)	0.02371 (15)	
S2A	0.29573 (10)	−0.30344 (6)	0.38262 (4)	0.02567 (16)	
S2B	0.22859 (9)	0.13688 (5)	1.07688 (4)	0.01906 (14)	
S3A	0.32482 (9)	−0.42492 (5)	0.56122 (4)	0.02351 (15)	
S3B	0.14624 (9)	0.23871 (5)	0.89130 (4)	0.02046 (15)	
O1A	0.2481 (3)	0.03651 (16)	0.55080 (11)	0.0221 (4)	
O1B	0.2523 (3)	−0.20849 (16)	0.90756 (12)	0.0243 (4)	
O1W	0.5774 (2)	0.02421 (15)	0.75431 (12)	0.0196 (4)	
N1A	0.2661 (3)	−0.07508 (18)	0.41864 (13)	0.0171 (4)	
H1AA	0.2675	−0.0709	0.3609	0.021*	
N1B	0.2789 (3)	−0.08541 (17)	1.04263 (13)	0.0149 (4)	
H1BA	0.3020	−0.0830	1.1018	0.018*	
N2A	0.3233 (3)	−0.17714 (18)	0.54658 (13)	0.0172 (4)	
H2AA	0.3276	−0.1065	0.5757	0.021*	
N2B	0.2633 (3)	0.02718 (18)	0.91718 (13)	0.0158 (4)	
H2BA	0.2896	−0.0347	0.8907	0.019*	
N3A	0.3823 (3)	−0.24320 (18)	0.69077 (14)	0.0198 (4)	
N3B	0.2608 (3)	0.10852 (18)	0.77715 (13)	0.0187 (4)	
C1A	0.1092 (4)	0.3120 (2)	0.36934 (18)	0.0230 (5)	
H1A	0.0858	0.3894	0.3665	0.028*	
C1B	0.2768 (4)	−0.5164 (2)	1.07967 (18)	0.0253 (5)	
H1B	0.2776	−0.5999	1.0804	0.030*	
C2A	0.0959 (4)	0.2312 (2)	0.29353 (18)	0.0235 (5)	
H2A	0.0615	0.2470	0.2331	0.028*	
C2B	0.2821 (4)	−0.4394 (2)	1.15643 (17)	0.0228 (5)	
H2B	0.2863	−0.4647	1.2153	0.027*	
C3A	0.1405 (3)	0.1203 (2)	0.31700 (17)	0.0196 (5)	
H3A	0.1388	0.0553	0.2738	0.024*	
C3B	0.2803 (3)	−0.3171 (2)	1.13637 (16)	0.0188 (5)	
H3B	0.2834	−0.2528	1.1805	0.023*	
C4A	0.1863 (3)	0.1199 (2)	0.41070 (16)	0.0163 (5)	
C4B	0.2735 (3)	−0.3040 (2)	1.04436 (16)	0.0164 (5)	
C5A	0.2352 (3)	0.0259 (2)	0.46643 (16)	0.0161 (5)	
C5B	0.2672 (3)	−0.1975 (2)	0.99201 (16)	0.0161 (5)	
C6A	0.2953 (3)	−0.1828 (2)	0.45404 (16)	0.0170 (5)	
C6B	0.2569 (3)	0.0239 (2)	1.00755 (16)	0.0152 (4)	
C7A	0.3461 (3)	−0.2706 (2)	0.60104 (17)	0.0167 (5)	
C7B	0.2328 (3)	0.1179 (2)	0.86155 (16)	0.0149 (4)	
C8A	0.3920 (4)	−0.3491 (2)	0.73281 (18)	0.0226 (5)	
H8A	0.4158	−0.3481	0.7967	0.027*	
C8B	0.2079 (4)	0.2002 (2)	0.73046 (17)	0.0210 (5)	
H8B	0.2173	0.2082	0.6691	0.025*	
C9A	0.3645 (4)	−0.4542 (2)	0.67459 (18)	0.0254 (6)	
H9A	0.3667	−0.5323	0.6932	0.030*	
C9B	0.1420 (4)	0.2768 (2)	0.77978 (17)	0.0219 (5)	
H9B	0.0998	0.3417	0.7570	0.026*	
H1W	0.486 (3)	0.052 (3)	0.750 (3)	0.050*	
H2W	0.531 (4)	−0.0536 (3)	0.744 (2)	0.050*	

### Atomic displacement parameters (Å^2^)

**Table:**

	*U*^11^	*U*^22^	*U*^33^	*U*^12^	*U*^13^	*U*^23^
S1A	0.0280 (3)	0.0159 (3)	0.0215 (3)	0.0091 (2)	0.0033 (2)	−0.0003 (2)
S1B	0.0384 (4)	0.0153 (3)	0.0203 (3)	0.0137 (3)	0.0053 (2)	−0.0009 (2)
S2A	0.0424 (4)	0.0177 (3)	0.0177 (3)	0.0141 (3)	0.0017 (3)	−0.0029 (2)
S2B	0.0280 (3)	0.0126 (3)	0.0167 (3)	0.0081 (2)	0.0033 (2)	−0.0014 (2)
S3A	0.0336 (3)	0.0113 (3)	0.0234 (3)	0.0059 (2)	0.0030 (2)	−0.0002 (2)
S3B	0.0286 (3)	0.0172 (3)	0.0218 (3)	0.0133 (2)	0.0095 (2)	0.0050 (2)
O1A	0.0312 (10)	0.0176 (8)	0.0176 (9)	0.0089 (7)	0.0043 (7)	−0.0004 (6)
O1B	0.0396 (11)	0.0183 (8)	0.0183 (9)	0.0139 (8)	0.0064 (8)	0.0011 (7)
O1W	0.0232 (9)	0.0117 (8)	0.0228 (9)	0.0067 (7)	0.0012 (7)	−0.0014 (7)
N1A	0.0231 (10)	0.0127 (9)	0.0151 (9)	0.0065 (8)	0.0015 (8)	0.0000 (7)
N1B	0.0183 (9)	0.0120 (9)	0.0145 (9)	0.0060 (7)	0.0019 (7)	0.0006 (7)
N2A	0.0217 (10)	0.0104 (9)	0.0175 (10)	0.0043 (7)	0.0018 (8)	−0.0020 (7)
N2B	0.0189 (9)	0.0123 (9)	0.0181 (10)	0.0079 (7)	0.0046 (8)	−0.0001 (7)
N3A	0.0210 (10)	0.0154 (10)	0.0204 (10)	0.0035 (8)	0.0015 (8)	0.0019 (8)
N3B	0.0229 (10)	0.0139 (9)	0.0192 (10)	0.0061 (8)	0.0042 (8)	0.0007 (8)
C1A	0.0238 (12)	0.0190 (12)	0.0285 (13)	0.0101 (10)	0.0045 (10)	0.0058 (10)
C1B	0.0376 (15)	0.0148 (11)	0.0254 (13)	0.0125 (10)	0.0030 (11)	0.0042 (10)
C2A	0.0228 (12)	0.0283 (13)	0.0220 (12)	0.0117 (10)	0.0037 (10)	0.0058 (10)
C2B	0.0313 (13)	0.0177 (12)	0.0184 (12)	0.0090 (10)	0.0006 (10)	0.0026 (9)
C3A	0.0189 (11)	0.0183 (11)	0.0220 (12)	0.0066 (9)	0.0043 (9)	0.0014 (9)
C3B	0.0210 (12)	0.0130 (11)	0.0202 (12)	0.0059 (9)	−0.0009 (9)	−0.0031 (9)
C4A	0.0141 (10)	0.0137 (10)	0.0195 (11)	0.0031 (8)	0.0023 (9)	−0.0008 (9)
C4B	0.0153 (10)	0.0117 (10)	0.0218 (12)	0.0055 (8)	0.0020 (9)	−0.0031 (9)
C5A	0.0139 (10)	0.0120 (10)	0.0199 (12)	0.0018 (8)	0.0022 (9)	−0.0015 (9)
C5B	0.0139 (10)	0.0124 (10)	0.0212 (12)	0.0045 (8)	0.0024 (9)	−0.0010 (9)
C6A	0.0164 (11)	0.0137 (10)	0.0182 (11)	0.0030 (8)	0.0002 (9)	0.0009 (8)
C6B	0.0132 (10)	0.0100 (10)	0.0201 (11)	0.0022 (8)	0.0009 (8)	0.0003 (8)
C7A	0.0150 (11)	0.0107 (10)	0.0219 (12)	0.0016 (8)	0.0026 (9)	−0.0003 (8)
C7B	0.0137 (10)	0.0109 (10)	0.0194 (11)	0.0042 (8)	0.0015 (8)	−0.0006 (8)
C8A	0.0228 (12)	0.0218 (12)	0.0217 (12)	0.0054 (10)	0.0026 (10)	0.0062 (10)
C8B	0.0245 (12)	0.0181 (11)	0.0196 (12)	0.0059 (10)	0.0029 (9)	0.0042 (9)
C9A	0.0304 (14)	0.0169 (12)	0.0269 (14)	0.0048 (10)	0.0036 (11)	0.0082 (10)
C9B	0.0268 (13)	0.0195 (12)	0.0219 (12)	0.0102 (10)	0.0042 (10)	0.0088 (10)

### Geometric parameters (Å, º)

**Table:**

S1A—C1A	1.709 (3)	N2B—H2BA	0.8600
S1A—C4A	1.727 (2)	N3A—C7A	1.306 (3)
S1B—C1B	1.705 (3)	N3A—C8A	1.379 (3)
S1B—C4B	1.725 (2)	N3B—C7B	1.303 (3)
S2A—C6A	1.655 (2)	N3B—C8B	1.382 (3)
S2B—C6B	1.657 (2)	C1A—C2A	1.362 (4)
S3A—C9A	1.721 (3)	C1A—H1A	0.9300
S3A—C7A	1.728 (2)	C1B—C2B	1.366 (4)
S3B—C7B	1.721 (2)	C1B—H1B	0.9300
S3B—C9B	1.726 (2)	C2A—C3A	1.418 (3)
O1A—C5A	1.231 (3)	C2A—H2A	0.9300
O1B—C5B	1.230 (3)	C2B—C3B	1.411 (3)
O1W—H1W	0.8199 (10)	C2B—H2B	0.9300
O1W—H2W	0.8199 (11)	C3A—C4A	1.370 (3)
N1A—C5A	1.387 (3)	C3A—H3A	0.9300
N1A—C6A	1.396 (3)	C3B—C4B	1.372 (3)
N1A—H1AA	0.8600	C3B—H3B	0.9300
N1B—C5B	1.383 (3)	C4A—C5A	1.463 (3)
N1B—C6B	1.393 (3)	C4B—C5B	1.461 (3)
N1B—H1BA	0.8600	C8A—C9A	1.347 (4)
N2A—C6A	1.344 (3)	C8A—H8A	0.9300
N2A—C7A	1.385 (3)	C8B—C9B	1.340 (4)
N2A—H2AA	0.8600	C8B—H8B	0.9300
N2B—C6B	1.348 (3)	C9A—H9A	0.9300
N2B—C7B	1.387 (3)	C9B—H9B	0.9300
			
C1A—S1A—C4A	91.39 (12)	C3A—C4A—C5A	131.8 (2)
C1B—S1B—C4B	91.37 (12)	C3A—C4A—S1A	111.53 (18)
C9A—S3A—C7A	88.14 (12)	C5A—C4A—S1A	116.61 (17)
C7B—S3B—C9B	88.16 (11)	C3B—C4B—C5B	131.9 (2)
H1W—O1W—H2W	106 (2)	C3B—C4B—S1B	111.50 (17)
C5A—N1A—C6A	127.1 (2)	C5B—C4B—S1B	116.60 (18)
C5A—N1A—H1AA	116.4	O1A—C5A—N1A	122.3 (2)
C6A—N1A—H1AA	116.4	O1A—C5A—C4A	121.5 (2)
C5B—N1B—C6B	126.7 (2)	N1A—C5A—C4A	116.1 (2)
C5B—N1B—H1BA	116.7	O1B—C5B—N1B	122.6 (2)
C6B—N1B—H1BA	116.7	O1B—C5B—C4B	121.1 (2)
C6A—N2A—C7A	128.3 (2)	N1B—C5B—C4B	116.3 (2)
C6A—N2A—H2AA	115.9	N2A—C6A—N1A	114.9 (2)
C7A—N2A—H2AA	115.9	N2A—C6A—S2A	125.44 (18)
C6B—N2B—C7B	128.1 (2)	N1A—C6A—S2A	119.68 (17)
C6B—N2B—H2BA	116.0	N2B—C6B—N1B	114.6 (2)
C7B—N2B—H2BA	116.0	N2B—C6B—S2B	125.81 (17)
C7A—N3A—C8A	110.1 (2)	N1B—C6B—S2B	119.57 (17)
C7B—N3B—C8B	109.9 (2)	N3A—C7A—N2A	118.5 (2)
C2A—C1A—S1A	112.31 (19)	N3A—C7A—S3A	115.58 (18)
C2A—C1A—H1A	123.8	N2A—C7A—S3A	125.85 (18)
S1A—C1A—H1A	123.8	N3B—C7B—N2B	118.3 (2)
C2B—C1B—S1B	112.34 (19)	N3B—C7B—S3B	115.79 (17)
C2B—C1B—H1B	123.8	N2B—C7B—S3B	125.78 (18)
S1B—C1B—H1B	123.8	C9A—C8A—N3A	115.1 (2)
C1A—C2A—C3A	112.5 (2)	C9A—C8A—H8A	122.4
C1A—C2A—H2A	123.7	N3A—C8A—H8A	122.4
C3A—C2A—H2A	123.7	C9B—C8B—N3B	115.3 (2)
C1B—C2B—C3B	112.4 (2)	C9B—C8B—H8B	122.3
C1B—C2B—H2B	123.8	N3B—C8B—H8B	122.3
C3B—C2B—H2B	123.8	C8A—C9A—S3A	111.08 (19)
C4A—C3A—C2A	112.3 (2)	C8A—C9A—H9A	124.5
C4A—C3A—H3A	123.9	S3A—C9A—H9A	124.5
C2A—C3A—H3A	123.9	C8B—C9B—S3B	110.83 (18)
C4B—C3B—C2B	112.4 (2)	C8B—C9B—H9B	124.6
C4B—C3B—H3B	123.8	S3B—C9B—H9B	124.6
C2B—C3B—H3B	123.8		
			
C4A—S1A—C1A—C2A	0.6 (2)	C7A—N2A—C6A—N1A	176.5 (2)
C4B—S1B—C1B—C2B	0.3 (2)	C7A—N2A—C6A—S2A	−4.3 (4)
S1A—C1A—C2A—C3A	−0.5 (3)	C5A—N1A—C6A—N2A	−10.2 (3)
S1B—C1B—C2B—C3B	−0.3 (3)	C5A—N1A—C6A—S2A	170.44 (18)
C1A—C2A—C3A—C4A	0.1 (3)	C7B—N2B—C6B—N1B	175.1 (2)
C1B—C2B—C3B—C4B	0.1 (3)	C7B—N2B—C6B—S2B	−5.7 (4)
C2A—C3A—C4A—C5A	177.8 (2)	C5B—N1B—C6B—N2B	−13.9 (3)
C2A—C3A—C4A—S1A	0.3 (3)	C5B—N1B—C6B—S2B	166.76 (18)
C1A—S1A—C4A—C3A	−0.49 (19)	C8A—N3A—C7A—N2A	177.4 (2)
C1A—S1A—C4A—C5A	−178.44 (19)	C8A—N3A—C7A—S3A	−0.9 (3)
C2B—C3B—C4B—C5B	179.2 (2)	C6A—N2A—C7A—N3A	176.3 (2)
C2B—C3B—C4B—S1B	0.1 (3)	C6A—N2A—C7A—S3A	−5.5 (4)
C1B—S1B—C4B—C3B	−0.2 (2)	C9A—S3A—C7A—N3A	0.9 (2)
C1B—S1B—C4B—C5B	−179.44 (19)	C9A—S3A—C7A—N2A	−177.3 (2)
C6A—N1A—C5A—O1A	7.0 (4)	C8B—N3B—C7B—N2B	174.97 (19)
C6A—N1A—C5A—C4A	−173.1 (2)	C8B—N3B—C7B—S3B	−1.3 (3)
C3A—C4A—C5A—O1A	−170.5 (2)	C6B—N2B—C7B—N3B	174.7 (2)
S1A—C4A—C5A—O1A	6.9 (3)	C6B—N2B—C7B—S3B	−9.5 (3)
C3A—C4A—C5A—N1A	9.6 (4)	C9B—S3B—C7B—N3B	1.54 (19)
S1A—C4A—C5A—N1A	−173.01 (16)	C9B—S3B—C7B—N2B	−174.4 (2)
C6B—N1B—C5B—O1B	6.8 (4)	C7A—N3A—C8A—C9A	0.5 (3)
C6B—N1B—C5B—C4B	−173.2 (2)	C7B—N3B—C8B—C9B	0.2 (3)
C3B—C4B—C5B—O1B	−176.5 (2)	N3A—C8A—C9A—S3A	0.2 (3)
S1B—C4B—C5B—O1B	2.5 (3)	C7A—S3A—C9A—C8A	−0.6 (2)
C3B—C4B—C5B—N1B	3.5 (4)	N3B—C8B—C9B—S3B	0.9 (3)
S1B—C4B—C5B—N1B	−177.49 (16)	C7B—S3B—C9B—C8B	−1.32 (19)

### Hydrogen-bond geometry (Å, º)

**Table:**

*D*—H···*A*	*D*—H	H···*A*	*D*···*A*	*D*—H···*A*
N1*A*—H1*AA*···O1*W*^i^	0.86	2.22	3.003 (3)	152
N1*B*—H1*BA*···O1*W*^ii^	0.86	2.14	2.973 (3)	163
N2*A*—H2*AA*···O1*A*	0.86	1.89	2.599 (3)	138
N2*B*—H2*BA*···O1*B*	0.86	1.90	2.588 (3)	136
O1*W*—H1*W*···N3*B*	0.82 (1)	2.06 (1)	2.852 (3)	163 (4)
O1*W*—H2*W*···N3*A*	0.82 (1)	2.09 (1)	2.892 (3)	167 (3)

Symmetry codes: (i) −*x*+1, −*y*, −*z*+1; (ii) −*x*+1, −*y*, −*z*+2.
